# Construct validity of the FOCUS^©^ (Focus on the Outcomes of Communication Under Six): a communicative participation outcome measure for preschool children

**DOI:** 10.1111/cch.12043

**Published:** 2013-06-13

**Authors:** K Washington, N Thomas-Stonell, B Oddson, S McLeod, G Warr-Leeper, B Robertson, P Rosenbaum

**Affiliations:** * Bloorview Research Institute, University of TorontoToronto, ON, Canada; † School of Human Kinetics, Laurentian UniversitySudbury, ON, Canada; ‡ Charles Sturt UniversityBathurst, NSW, Australia; § School of Communication Sciences and Disorders, University of Western OntarioLondon, ON, Canada; ¶ Bloorview Research InstituteToronto, ON, Canada; ** CanChild Centre for Childhood Disability Research, McMaster UniversityHamilton, ON, Canada

**Keywords:** communicative participation, construct validity, FOCUS^©^, outcome measures, preschoolers, speech-language intervention, World Health Organization

## Abstract

**Objective:**

The aim of this study was to establish the construct validity of the Focus on the Outcomes of Communication Under Six (FOCUS^©^). This measure is reflective of concepts in the International Classification of Functioning Disability and Health – Children and Youth framework. It was developed to capture ‘real-world’ changes (e.g. communicative participation) in preschoolers' communication following speech-language intervention.

**Method:**

A pre–post design was used. Fifty-two parents of 3- to 6-year-old preschoolers attending speech-language therapy were included as participants. Speech-language therapists provided individual and/or group intervention to preschoolers. Intervention targeted: articulation/phonology, voice/resonance, expressive/receptive language, play, and use of augmentative devices. Construct validity for communicative participation was assessed using pre-intervention and post-intervention parent interviews using the FOCUS^©^ and the communication and socialization domains of the Vineland Adaptive Behavior Scales-II (VABS-II).

**Results:**

Significant associations were found between the FOCUS^©^, measuring communicative participation, and the VABS-II domains for: (i) pre-intervention scores in communication (*r* = 0.53, *P* < 0.001; 95% CI 0.30–0.70) and socialization (*r* = 0.67, *P* < 0.001; 95% CI 0.48–0.80); (ii) change scores over-time in communication (*r* = 0.45, *P* < 0.001; 95% CI 0.201–0.65) and socialization (*r* = 0.39, *P* = 0.002; 95% CI 0.13–0.60); and (iii) scores at post-intervention for communication (*r* = 0.53, *P* < 0.001; 95% CI 0.30–0.70) and for socialization (*r* = 0.37, *P* = 0.003; 95% CI 0.11–0.50).

**Conclusions:**

The study provided evidence on construct validity of the FOCUS^©^ for evaluating real-world changes in communication. We believe that the FOCUS^©^ is a useful measure of communicative participation.

## Introduction

With the advent of evidence-based practices and the initiative to improve the quality of services, it has become increasingly important to measure clients' outcomes (Jette & Haley 2005; Cheung *et al*. 2012; King *et al*. 2012; Post *et al*. 2012). The growing need to generate evidence to guide clinical practices has fostered a demand for the development of feasible and sensitive measures to monitor the outcomes of interventions (Cheung *et al*. 2012). Outcome measures assess the end-results of health service interventions and programs. These tools evaluate changes in function following the implementation of some type of intervention. Using numerical terms, an outcome measure can establish the impact of intervention on clients' lives (Patrick & Chiang 2000).

To guide the measurement of outcomes, a theoretical framework is needed (Post *et al*. 2012). For professions within the field of developmental disabilities, the World Health Organization (WHO)'s framework, the International Classification of Functioning, Disability and Health – Children and Youth (ICF-CY) (WHO 2007) provides a theoretical context for conceptualizing children's Body Functions and Structures (e.g. mental functions of language, articulation functions), Activities and Participation (e.g. ability to execute tasks or actions in everyday life situations), and Environmental and Personal Factors (McLeod & McCormack 2007; Washington 2007, 2010; McLeod & Threats 2008). As a result, outcomes of interventions at these several levels should be assessed (Jette & Haley 2005).

In speech-language pathology, there is a paucity of measures that evaluate Activities and Participation outcomes, namely communicative participation, defined as ‘communication in life situations where knowledge, information, ideas, or feelings are exchanged’ (Eadie *et al*. 2006; Yorkston *et al*. 2008) and includes using communication skills to be included with others (e.g. using communication skills to join in conversations with others to play a game, at meal time, or at the zoo). This paucity is not unique to speech-language pathology, as it also extends to other rehabilitation professions (Djijker *et al*. 2000; Post *et al*. 2012). However, there is a critical and growing need to measure this outcome level, which describes the clients' abilities use their current level of functioning to be included with others (Eadie 2003).

The available outcome measures for speech-language pathology [e.g. the American Speech-Language-Hearing Association Pre-Kindergarten National Outcome Measure System (ASHA Pre-K NOMS; NOMS 2000); Therapy Outcome Measure (TOM; Enderby 1997); and the AusTOMS (the Australian adaptation of the TOMS; Perry *et al*. 2004)] are aligned with the previous WHO frameworks that do not have a specific focus on children and youth and do not focus on communicative participation changes for this population (Thomas-Stonell *et al*. 2007). In contrast, the Speech Participation and Activity Assessment of Children (SPAA-C; McLeod 2004) does provide an evaluation of Activity and Participation for children, is based on the ICF, and considers communication performance with others (e.g. teachers, parents, siblings) (McLeod *et al*. 2012). However, the SPAA-C does not provide numerical scores that can be used to consider outcomes, and the focus of the SPAA-C is on children with speech impairment and not the broader group of children with speech and language impairments. The dearth of communicative participation outcome measures limits speech-language pathologists' (SLPs) knowledge about potential ‘real-world’ changes in communication skills following speech-language intervention.

For preschoolers with communication disorders, there are negative consequences on the formation and maintenance of socially productive relationships and on later academic skills (Catts *et al*. 2002; Hart *et al*. 2004). Since SLPs work to ‘facilitate the preschooler's activities and participation by assisting the child to acquire new communication skills and strategies’ (ASHA 2004), establishing communicative participation outcomes following intervention is essential.

A new measure of communicative participation, reflective of the ICF-CY framework, has been developed. The *Focus on the Outcomes of Communication Under Six* (FOCUS^©^) is designed to capture real-world changes (e.g. using words and sentences to join in conversations with others) in children's communication associated with speech-language intervention (Thomas-Stonell *et al*. 2009). Unlike the SPAA-C (McLeod 2004), the FOCUS^©^ was designed to be used with a variety of children with speech and language impairments, and not only those identified with speech impairments. Similar to the SPAA-C, the FOCUS^©^ considers communication with others (i.e. Activity and Participation) for the purpose of being included in play and other activities. The reliability of the FOCUS**^©^** is established (Thomas-Stonell *et al*. 2009; Washington *et al*. 2013). The next step is to ensure that the measure does what it is intended to do, that is, establish its construct validity. Construct validity is the extent to which a measure correlates with the construct it was designed to measure (Streiner & Norman 2008). Typically, a number of independent studies are needed to establish the credibility of a measure (Unsworth *et al*. 2004).

## Aims

This study investigated the construct validity of the FOCUS^©^ for changes in communicative participation in a variety of preschoolers with speech-language impairments, including those with dual diagnoses. Associations between the FOCUS^©^ and a ‘gold-standard’ interview-based tool that considers communicative participation (e.g. socialization) in a wide range of young children needed to be completed. The Vineland Adaptive Behavior Scales II (VABS-II; Sparrow *et al*. 2005) is a valid and reliable interview-based standardized assessment of everyday adaptations for four major domains, including Communication, Daily Living Skills, Socialization and Motor Skills, for individuals from birth to 90 years of age. The VABS-II is also an established measure purported to monitor progress in level of functioning following intervention. Previous studies with children with communication disorders (e.g. Baker-Ericzén *et al*. 2007; Washington 2013) have used the Vineland test as an outcome measure to establish change over time associated with intervention. The VABS-II was chosen as a comparison instead of a speech and language measure (e.g. the TOM, AusTOMS, ASHA Pre-K NOMS, SPAA-C) because it concerns participation and function in daily life in a variety of individuals, including young children. Further, like the FOCUS^©^, the VABS-II uses an interview-based approach.

Associations (i.e. convergent validity) between the FOCUS^©^ and the Socialization and Communication domains of the VABS-II were investigated. The communication domain of the VABS-II was utilized because skills in communication are needed for participation (Hart *et al*. 2004). A moderate correlation was hypothesized because the VABS-II and the FOCUS^©^ measure similar, not identical constructs. The VABS-II measures broader Socialization (e.g. interpersonal skills, play, and coping) and Communication (e.g. receptive, expressive, and written language). Alternately, the FOCUS^©^ measures a subset of participation skills as they relate to communication, communicative participation (e.g. can respond to questions during a conversation).

## Methods

### Study design and sampling

This study used a pre–post design. Three Ontario, Canada organizations providing funded access to speech-language therapy for preschoolers participated. Following ethical approval from each organization, SLPs invited parents/caregivers on their caseload with children 6-years-old or younger to participate. The following inclusion criteria were used: (i) child identified with a speech and/or language impairment by registered SLPs; (ii) child enrolled in speech-language therapy from one of the three participating organizations; and (iii) SLP report of parental English proficiency. Informed consent was obtained from 52 (50 mothers, 2 fathers) of the 81 parents invited to participate. Power analyses were conducted and for convergent validity, it was estimated that 32 or more participants would be adequate for the hypothesized correlations.

### Demographics

Preschool participants (*n* = 52) ranged in age from 3 years 1 month to 6 years 0 months (mean = 4 years 6 months; SD = 8.04 months), of whom thirty-two were boys (62%). The most commonly identified speech-language impairments were: speech and language disorder only (64%), language disorder only (21%), and speech sound disorder only (15%). Twenty-four (46%) of the participants had a specific medical diagnosis, including cerebral palsy (*n* = 14), hypotonia (*n* = 3), clubfoot (*n* = 2), global developmental delay (*n* = 2), spina bifida (*n* = 2) and autism spectrum disorder (*n* = 1). The current project was a measurement validation study that deliberately included a heterogeneous range of children with functional/medical limitations and communication disorders.

Therapy goals across preschoolers were: Articulation/Phonology (33%), Expressive Language (29%), Receptive Language (14%), Intelligibility (14%), Voice/Resonance (5%), Social/Play (3%), and use of Augmentative and Alternative Communication Devices (2%). Each preschooler's intervention was reflective of current community-based practices for each organization. On average, preschoolers received 16 h of direct group or individual intervention with a SLP (range = 3–57 h, inter-quartile range = 11.4). Individual intervention was provided 65% of the time, group intervention was provided 25% of the time, and group plus individual intervention was provided 10% of the time. Intervention frequency varied from once per week to three times per week; however, consistent with current service delivery models in the participating organizations, most preschoolers (79%) received intervention once weekly.

### Materials

The FOCUS^©^ is a newly developed measure of communicative participation, measuring changes in these skills (Thomas-Stonell *et al*. 2009). For the original development of the FOCUS^©^, information on children's abilities to communicate and participate in his or her community was gathered for 375 preschoolers. These preschoolers were identified with speech and/or language impairments, with or without concomitant medical diagnoses (e.g. cerebral palsy).

The FOCUS^©^ contains 50 items reflective of the ICF-CY framework (e.g. Body Functions and Structures, Activities and Participation, and Personal Factors), with most items capturing changes in Activities and Participation, i.e. ability to execute tasks or actions in everyday life situations, (cf. Thomas-Stonell *et al*. 2009, 2009; (see Appendix). Changes in children's communicative participation for both capacity (what the child is capable of doing in an ideal ‘therapeutic’ environment) and performance (what child is able to do in daily environments such as home, daycare) (cf. Thomas-Stonell *et al*. 2009, 2009) are evaluated. The FOCUS^©^, which can be administered independently by a SLP in a parent-interview format (or self-completion by parent/caregiver) in 10 min, is a criterion-referenced measure where performance is judged based on pre-established criteria (Thomas-Stonell *et al*. 2009). Information obtained from the FOCUS^©^ provides a ‘snapshot’ of the child's skill. During the FOCUS^©^ administration, parents responded to statements about their child's abilities with others in meaningful ways (e.g. ‘My child makes friends easily’) on a 7-point scale from ‘not at all like my child’ to ‘exactly like my child’ or ‘can always do without help’ to ‘cannot do at all’.

The VABS-II is an established norm-referenced measure of Socialization and Communication that has good psychometric properties and can be used for progress monitoring of a client's level of functioning (Sparrow *et al*. 2005). For the VABS-II a representative sample of over 3000 individuals, including a wide range of children with a broad range of medical conditions (e.g. Down Syndrome, orthopaedic impairments) and/or disorders in areas like communication and socialization were used. This measure was designed to be administered by individuals with graduate-level training in psychology or social work (Sparrow *et al*. 2005) and, therefore, should not be administered and interpreted independently by a SLP. In this study, the SLP completing the VABS-II did so under the supervision of a licensed psychologist. The VABS-II can be administered in 20–60 min. During the VABS-II interview portion, parents provided descriptions about their child's socialization and communication. For the socialization domain, parental descriptions were grouped according to three sub-domains: (i) Interpersonal Relationships – how their child interacts with others (e.g. ‘How does Johnny initiate play with other kids’); (ii) Play and Leisure Time – how does their child spend his or her play and leisure time (e.g. ‘How does Johnny play with toys/objects?’); and (iii) Coping – how does their child demonstrate sensitivity and responsibility to others (e.g. ‘How does Johnny respond to a change in his routine?’). For the communication domain, parental descriptions were grouped according to three sub-domains: (i) Receptive, i.e. how their child understands, listens and attends, and follows instructions (e.g. ‘How long will Johnny listen to a story?’); (ii) Expressive, i.e. how their child expresses himself or herself (e.g. ‘How does Johnny tell someone how to play a game?’); and (iii) Written, i.e. how is their child's beginning to read, reading and writing skills (e.g. ‘When you ask Johnny to identify specific alphabet letters, what does he do?’). Parents' responses were scored using the following options: (i) usually – ‘2’, (ii) sometimes or partially – ‘1’, (iii) never – ‘0’, or (iv) don't know.

### Procedure

Parents completed 40-min interviews about their child's participation and communication skills pre-intervention (admission) and post intervention (discharge) with an independent SLP who was not involved in the children's intervention. Within each pre- and post-intervention session, interviews were completed using the FOCUS^©^ and the Socialization and Communication domains of the VABS-II. Administration of the VABS-II and the FOCUS^©^ was counterbalanced across participants and phases.

### Statistical analysis/design

A pre–post intervention design was utilized. The construct validity of the FOCUS^©^ was evaluated using bivariate Pearson correlations with one-tailed significance tests. The use of one-tailed tests was appropriate since there was an expectation for scores on the FOCUS^©^ and the VABS-II domains to be positively correlated (e.g. positive change scores). This meant that the direction for performance was such that children's Communicative Participation, as measured by the FOCUS^©^, and Socialization and Communication, as measured by the VABS-II, would improve, not decline over time, following speech-language intervention. Raw scores at pre-intervention, post-intervention and raw change scores for pre- to post-intervention assessments on the FOCUS^©^ and the VABS-II total Socialization and Communication scale were calculated and entered in the correlational analysis. Raw scores were entered into the Statistical Program for the Social Sciences (PASW Statistics 2009) to complete the correlational analysis. To classify the size of the observed relationships, guidelines from Cohen (1988) were used where 0.10–0.30 = weak, 0.30–0.50 = moderate, >0.50 = high. An alpha level of 0.05 was utilized to establish statistical significance (Perneger 1998). There was no study attrition or missing data.

## Results

### FOCUS^©^ and the VABS-II associations

On average, children's scores improved from pre-intervention to post intervention on both the FOCUS^©^ and the two domains of the VABS-II. Mean scores and mean change scores along with standard deviations for both measures at each time point and over time are in Table 1.

**Table 1:** Means and standard deviations for VABS-II and FOCUS*©* scores

Measure	*N* (sample size)	Pre (raw score)	Post (raw score)	Pre-to-post (change raw score)
VABS-II Socialization Domain	52	Mean = 119.2	Mean = 144.4	Mean = 25.1
		SD = 22.7	SD = 17.6	SD = 21.0
VABS-II Communication Domain	52	Mean = 113.9	Mean = 139.1	Mean = 25.1
		SD = 24.7	SD = 18.3	SD = 18.0
FOCUS^©^	52	Mean = 253.8	Mean = 290.4	Mean = 36.5
		SD = 51.5	SD = 40.1	SD = 36.1

FOCUS^©^, Focus on the Outcomes of Communication Under Six (Thomas-Stonell *et al*. 2009); VABS-II, Vineland Adaptive Behavior Scales (Sparrow *et al*. 2005); pre, pre-intervention; post, post-intervention.

Correlations with 95% confidence intervals between the FOCUS^©^ and the VABS-II domains are in Table 2. To evaluate the construct validity of the FOCUS^©^, raw scores and raw change scores obtained from the FOCUS^©^ and VABS-II were utilized.

**Table 2:** Correlations between the FOCUS^©^ and the VABS-II

Measures	Time point	Correlation	95% CI
VABS-Comm FOCUS^©^	Pre	0.53*	0.30–0.70
VABS-Soc FOCUS^©^	Pre	0.67*	0.48–0.80
VABS-Comm FOCUS^©^	Post	0.53*	0.30–0.70
VABS-Soc FOCUS^©^	Post	0.37**	0.11–0.50
VABS-Comm FOCUS^©^	Pre-to-post	0.45*	0.20–0.65
VABS-Soc FOCUS^©^	Pre-to-post	0.39**	0.13–0.63

* *P* < 0.001 (statistically significant beyond *P* < 0.05).

** *P* < 0.01 (statistically significant beyond *P* < 0.05).

CI, confidence interval; FOCUS^©^, Focus on the Outcomes of Communication Under Six (Thomas-Stonell *et al*. 2009); VABS-Comm, Communication Domain of the Vineland Adaptive Behavior Scales; VABS-II, Vineland Adaptive Behavior Scales (Sparrow *et al*. 2005); VABS-Soc, Socialization Domain of the Vineland Adaptive Behavior Scales; pre, pre-intervention; post, post-intervention.

### Communication skills

There was a significant positive correlation between scores on the FOCUS^©^ and the VABS-II Communication domain at the beginning of intervention (*r* = 0.53, *P* < 0.001; 95% CI 0.30–0.70, *n* = 52) and at the end of the intervention period (*r* = 0.53, *P* < 0.001; 95% CI 0.30–0.70, *n* = 52). Further, the FOCUS^©^-measured changes over time were positively correlated with changes on the VABS-II (*r* = 0.45, *P* < 0.001; 95% CI 0.21–0.65, *n* = 52).

### Communicative participation skills

There was a significant positive association between FOCUS^©^ and VABS-II Socialization domain total scores at pre-intervention (*r* = 0.67, *P* < 0.001; 95% CI 0.48–0.80, *n* = 52). A significant positive association was also found between total scores at post intervention (*r* = 0.37, *P* = 0.003; 95% CI 0.11–0.504, *n* = 52), suggesting a similar pattern to the pre-intervention association. It was also found that the amount of change over time as measured by the FOCUS^©^ was positively correlated with VABS-II Socialization domain change scores (*r* = 0.39, *P* = 0.002; 95% CI 0.128–0.597, *n* = 52).

## Discussion

This study was motivated by the continuing need to develop valid and reliable outcome measures that capture changes in a client's functioning after intervention. Following the development of the FOCUS^©^, previous investigations about its reliability were successfully completed (Thomas-Stonell *et al*. 2009; Washington *et al*. 2013). The construct of communicative participation, however, had yet to be investigated. The current study sought to address the validity of the FOCUS^©^ for this construct.

### Pre-intervention and post-intervention scores

The significant correlations at pre-intervention showed that both the FOCUS^©^ and the VABS-II captured children's current functioning. The higher scores on both measures suggested that as children became better at communicative participation, they were better able to use their communication skills during social interactions with others. The fact that this progression was observed on both measures suggests that the FOCUS^©^ can capture increased communicative participation.

### Change scores

Change scores were calculated for children's performance over time. The amount of change as measured by the FOCUS^©^ was moderately and positively correlated with changes on the VABS-II, Socialization and Communication domains. The positive associations between the two VABS-II domains and the FOCUS^©^ suggest that the FOCUS^©^ is sensitive to changes in children's communicative participation.

### Correlations between measures

Moderate to high positive correlations were found between the FOCUS^©^ and the VABS-II, Communication and Socialization domains for scores at pre-intervention, post-intervention, and change scores pre-intervention to post-intervention. The FOCUS^©^ appears to be sensitive to young children's current functioning as well as change in functioning as it relates to communicative participation following speech and language intervention. This finding supports the construct (convergent) validity of the FOCUS^©^.

Based on the current findings, it is evident that the FOCUS^©^ has clinical utility for speech-language pathology. These findings support the notion that when SLPs work to facilitate children's acquisition of new communication skills during speech and language intervention, corresponding improvement in these children's activities and participation are evident following intervention.

## Limitations

One limitation of this study was that an available sample of parents of children with communication disorders was recruited. Since this sample of parents was based on the caseload at three centres, the children may not be completely representative of all children identified with communication disorders. Further, the age range of this sample is restricted compared with the planned sample for the FOCUS^©^ as there were no children less than 3 years old. The sample of available children included was, however, randomly selected to be included in the study and was reflective of the participant sample used in the development of both the FOCUS^©^ and the VABS-II. Consequently, the sample of children does match the target populations that both measures purport to assess.

To reduce respondent burden, the complete VABS-II was not administered. The lack of information on divergent validity of the FOCUS^©^ is acknowledged as another limitation. That said, validity studies published by the group of researchers who established validity of the AusTOMs scales also examined construct (convergent) validity, without simultaneously reporting on divergent validity (Unsworth *et al*. 2004). Therefore, this study is consistent with other studies engaged in the development of outcome measures. Other researchers engaged in measurement development state, ‘the validity of a tool is never fully confirmed’ (Unsworth *et al*. 2004, p. 74). Instead, a series of studies is required to ensure that a measure does what it is intended to do (Streiner & Norman 2008). Future work could investigate the ability of the FOCUS^©^ to predict changes in communicative participation and to also discriminate amount of change amongst children with differing levels of speech-language functioning. An additional next step is to be able to convert FOCUS^©^ scores to specific ICF-CY codes to help track overall outcomes for children.

## Conclusion

Measuring changes in communicative participation adds an important dimension to the measurement of therapeutic outcomes in speech-language intervention. The FOCUS^©^ is a measure of participation, evaluating one construct, communicative participation. Unlike the VABS-II, the FOCUS^©^ can be administered and interpreted independently by an SLP and is a criterion-referenced measure, thus sensitive to changes at the individual level. The FOCUS^©^ (i) has a shorter administration time; (ii) requires little training; (iii) can be self-completed by parents/caregivers of children with communication disorders; and (iv) captures current communicative participation and changes in communicative participation, a construct of keen importance for paediatric speech-language pathology.

Quality in healthcare services provided to young children with communication disorders can be improved by using outcome measures that are feasible, sensitive and valid. This study provides evidence that the FOCUS^©^ is an appropriate change-detecting tool for use in speech-language pathology.

**Key messages**

- The evaluation of communicative participation is needed within rehabilitation professions like speech-language pathology.
- The FOCUS^**©**^ is a valid change-detecting tool, useful for evaluating communicative participation changes for preschoolers with communication disorders.
- This tool contains 50 items that are consistent with the ICF-CY framework.
- The FOCUS^**©**^ provides speech-language pathologists with an easy-to-administer tool for evaluating communicative participation outcomes following paediatric speech and language intervention.

## Appendix

### Focus on the Outcomes of Communication Under Six (FOCUS^©^) items

http://www.hollandbloorview.ca/research/FOCUS/FOCUS_works.php

**Table:**

Part 1	FOCUS^©^ item
1.	My child makes friends easily.
2.	My child is included in play activities by other children.
3.	My child is comfortable when communicating.
4.	My child is confident communicating with adults who know my child well.
5.	My child takes turns.
6.	My child talks while playing.
7.	My child is willing to talk to others.
8.	My child is confident communicating with adults who do not know my child well.
9.	My child can communicate independently.
10.	My child talks a lot.
11.	My child can string words together.
12.	My child gets along with other children.
13.	My child can communicate independently with other children.
14.	My child's speech is clear.
15.	My child understood the first time when s/he is talking with other children.
16.	My child speaks slowly when not understood.
17.	My child speaks in complete sentences.
18.	My child uses communication to solve problems.
19.	My child waits for her/his turn to talk.
20.	My child conveys his/her ideas with words.
21.	My child uses correct grammar when speaking.
22.	My child uses new words.
23.	My child uses words to ask for things.
24.	My child's communication skills get in the way of learning.
25.	My child's communication skills limit her/his independence.
26.	My child is understood the first time when talking with adults who do not know my child well.
27.	My child can tell adults who do not know my child well about past events.
28.	My child uses language to communicate new ideas.
29.	My child needs help to be understood by other children.
30.	My child becomes frustrated when trying to communicate with other children.
31.	My child can communicate independently with adults who do not know my child well.
32.	My child is reluctant to talk.
33.	My child can talk to other children about what s/he is doing.
34.	My child has difficulties changing activities.

**Table:**

Part 2	FOCUS^©^ item
1.	My child plays well with other children.
2.	My child will sit and listen to stories.
3.	My child can communicate effectively with adults who know my child well.
4.	My child is included in games by other children.
5.	My child will try to carry on a conversation with adults who do not know my child well.
6.	My child will ask for things from adults s/he knows well.
7.	My child participates in group activities.
8.	My child can tell stories that make sense.
9.	My child can respond to questions.
10.	My child will ask for things from other children.
11.	My child can carry on a conversation with other children.
12.	My child can communicate effectively with other children.
13.	My child can communicate effectively with adults who do not know my child well.
14.	My child can be understood by other children.
15.	My child can talk about what s/he is doing with adults who do not know my child well.
16.	My child joins in conversations with her/his peers.
